# TinyML: Enabling of Inference Deep Learning Models on Ultra-Low-Power IoT Edge Devices for AI Applications

**DOI:** 10.3390/mi13060851

**Published:** 2022-05-29

**Authors:** Norah N. Alajlan, Dina M. Ibrahim

**Affiliations:** 1 Department of Information Technology, College of Computer, Qassim University, Buraydah 51452, Saudi Arabia; 411200285@qu.edu.sa; 2 Department of Computers and Control Engineering, Faculty of Engineering, Tanta University, Tanta 31733, Egypt

**Keywords:** Internet of Things, edge devices, machine learning, deep learning, tiny machine learning

## Abstract

Recently, the Internet of Things (IoT) has gained a lot of attention, since IoT devices are placed in various fields. Many of these devices are based on machine learning (ML) models, which render them intelligent and able to make decisions. IoT devices typically have limited resources, which restricts the execution of complex ML models such as deep learning (DL) on them. In addition, connecting IoT devices to the cloud to transfer raw data and perform processing causes delayed system responses, exposes private data and increases communication costs. Therefore, to tackle these issues, there is a new technology called Tiny Machine Learning (TinyML), that has paved the way to meet the challenges of IoT devices. This technology allows processing of the data locally on the device without the need to send it to the cloud. In addition, TinyML permits the inference of ML models, concerning DL models on the device as a Microcontroller that has limited resources. The aim of this paper is to provide an overview of the revolution of TinyML and a review of tinyML studies, wherein the main contribution is to provide an analysis of the type of ML models used in tinyML studies; it also presents the details of datasets and the types and characteristics of the devices with an aim to clarify the state of the art and envision development requirements.

## 1. Introduction

Over the last decade, we have seen the development of machine learning algorithms with the development of the Internet of Things (IoT) as in microelectronics, communication, and information technology. The IoT concept has been widely used in different aspects of our lives with applications and technologies [1,2] including smart cities, smart environments, smart homes, etc. Billions of IoT devices are connected to the internet; with extensive numbers of IoT devices comes the mass production of IoT platforms [1,3]. The role of these devices is to sense the physical features of their deployment environments—twenty-four hours a day, seven days a week. This leads to a rise in the volume of data generated that in turn needs high computing performance and large amounts of storage space. Recently, the integration of Machine Learning (ML) algorithms with the IoT devices has aimed to process huge amounts of data and make the devices intelligent in order to make decisions [3].

Deep neural networks (DNNs) or deep learning (DL) is a subset of ML and considers the most advanced algorithms to process data. In recent years DL has witnessed rapid development in successful applications in different areas, e.g., image classification, object detection and speech recognition. Meanwhile, DL enables many applications in IoT edge devices such as mobiles, which become intelligent microcontrollers once equipped with DL, e.g., Apple Siri to enable human-computer interactions [4]. Efforts have been made to deploy DL into IoT devices, due to advantages in terms of reducing response time latency, bandwidth connection, energy consumption, security, and privacy [5]. IoT edge devices can collect data using only the device itself, take action and make decisions based on the data capture. Local processing can be performed once a running pre-trained DL-model capable of making inferences is added into the device. In addition, some data is sent to micro-cloud computing, e.g., fog computing to perform processing before returning results to the device. However, inference of DL in edge devices is not yet prepared enough to be fully executed [3,4,6].

Many challenges involved in integrating DL with edge devices are listed here. (i) Training DL models is the most difficult challenge involved in integrating edge devices with DL, wherein the training DL models consist of dense parameters that constitute heavy weight to achieve high accuracy. It is computationally expensive, consuming massive CPU and GPU resources, power, memory, and time. However, edge devices have not yet become cost-efficient enough to take training DL models due to limited resources [7,8,9]. For instance, in [10] it is shown that the training of DL is the main obstacle in integrating DL with the industries internet of things (IIoT), due to the complications of DL models which take up time in the training phase. (ii) Inference of DL in edge devices is a significant challenge; based on the complicated DL models, training inference in DL models takes a long time and can cause delays in response times.

Currently, DL is partially deployed on edge devices and the remaining data are transferred to processing in the cloud, or DL models are deployed in the cloud to process raw data that are received from edge devices, thus causing a delay in latency [9]. For instance, Ref. [11] edge devices are used to sense water data and then transfer the data to the cloud to analyze; data can also be forecast using DL models. (iii) Power and memory consumption are present challenges; heavy-weight DL models consume memory and a lot of power. The size of memory and power capacity in edge devices is limited, since these devices have small memory and short energy lifetimes. Thus, the performance of DL models will be significantly affected compared to the servers in data centers or devices that have large resource as power and memory [12]. (iv) Security constitutes a challenge to integrating DL with edge devices. The proliferation of IoT edge devices lead to collection of sensitive data from society, wherein transmission of data to the cloud may expose it to hacking and eavesdropping [13].

**Figure 1:**
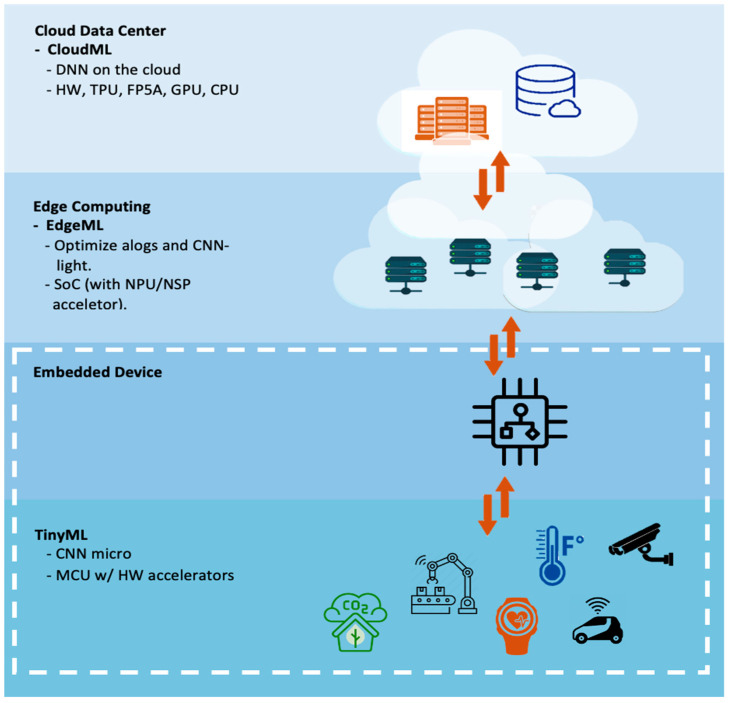
A framework of IoT applications with Cloud computing, Edge computing and TinyML.

A new concept has emerged of a meeting point and intersection between machine learning (deep learning) and an edge device called TinyML. TinyML enables deploy of small DL models into a tiny edge device that has tough resource constraints e.g., limited computation (clock speed about tens of megahertz), small memory and a few milliwatts (mW) of power. TinyML allows analysis and interpretation of data locally on the devices and takes action in real time [14]. Furthermore, deployment of pre-trained DL models into tiny edge devices is now possible, after performing some techniques to compress DL models and optimize the inference. For instance, using quantization techniques, which are conversion techniques that convert float-point numbers to minimize precision numbers, intending to shrink the size of the DL model with minimal degradation of accuracy. Pruning techniques allow removal of redundant structures of network and parameters [15,16]. Figure 1 depicts the capability of TinyML to process the data from various IoT devices locally into tiny edge devices (e.g., a microcontroller) without the need to connect to the cloud to process the data. However, TinyML has many advantages that can translate into saving huge costs, energy, and better protection of privacy. Details of TinyML will be mentioned in the next section.

The main contribution of this paper is to review the emerging topic of TinyML and their techniques in order to support the researchers in this field. The contributions are listed as follows:

(i) TinyML studies are reviewed in two aspects; first, studies that have developed the DL model and applied it in IoT applications. Second, studies that design frameworks and libraries for TinyML.
(ii) Analysis and findings from previous studies are provided for the main three items used in TinyML (Model, Dataset and Devices).
(iii) Discussion of main findings in TinyML (Model, Dataset and Devices) is presented.
(iv) Light is shed on the most relevant limitation concerning TinyML, which will provide the directions for future research.

The rest of the paper is organized as follows. Section 2 presents an overview of TinyML with mentions of the advantages of TinyML. In Section 3, our research methodology approach is demonstrated. Section 4 summarizes the related work of TinyML. Section 5 discusses the findings of the dataset and devices of TinyML studies. Section 6 contains analysis of the limitations of TinyML approaches. Finally, Section 7 illustrates the conclusions.

## 2. Overview of TinyML

The Tiny Machine Learning (TinyML) is an emerging field that has resulted in many inventions and is leading to the rapid growth of IoT fields e.g., smart manufacturing, smart health, autonomous driving, etc. TinyML is an alternative paradigm that allows implementing DL tasks locally on ultra-low-power devices, typically under a milliWatt. Thus, it allows for real-time analysis and interpretation of data, which translates to massive advantages in terms of latency, privacy, and cost [17,18].

The primary goal of TinyML is to improve the adequacy of DL systems through requiring less computation and less data, which will facilitate the giant market of edge AI and the IoT [17]. According to the universal tech market advisory company, ABI Research, a total of 2.5 billion devices are expected to be shipped with a TinyML chipset in 2030. These devices focus on advanced automation, low cost, low latency in transmitting data, and ultra-power-efficient Artificial Intelligence (AI) chipsets. The chipsets are known as edge AI or embedded AI, since they perform AI inference almost fully on the board, whereas in the training phase for these devices it will continue to depend on the external resources, such as gateways, on-premises servers, or the cloud.

Recently, TinyML has attracted the interest of industry giants. For instance, Google has released the TensorFlow Lite platform which allows the running of Neural Network (NN) models on IoT devices [19]. Likewise, Microsoft has released EdgeML [20], whereas ARM [21] has published an open-source library for Cortex-M processors that increase the performance of NN, known as Cortex Microcontroller Software Interface Standard Neural Network (CMSIS-NN). In addition, there is a new package called X-Cube-AI [22], which was released to execute DL models on STM 32-bit microcontrollers [5].

Microcontroller units (MCUs) are considered ideal hardware platforms for TinyML, due to typically being small (~1 cm^3^), low power (~1 mW), and cheap (~$1). MCUs combine a CPU, digital and analog peripherals on the chip embedded flash (eFlash) memory in order to store the program, in addition to the Static Random-Access Memory (SRAM) for intermediate data [17].

### Benefits of TinyML

In this section, a series of benefits of TinyML are mentioned that exceed the possible cons when integrated with IoT devices, especially with edge or daily use devices:

- Energy efficiency: this is a great benefit when adopting TinyML that works on MCUs. Since IoT devices that work on MCUs rely on batteries or even energy harvesting, they consume less energy in comparison with powerful processors and Graphics Processing Units (GPUs) that demand a great amount of power. Therefore, IoT devices can be placed everywhere without the need to be plugged into the power grid. Thus, it opens the door for novel cognitive itinerant applications. In addition, scarce power consumption allows the IoT devices to be coupled with larger battery-powered devices, hence converting them into connected smart entities, e.g., personal mobility devices such as scooters or seaways [23].
- Low cost: IoT devices performed a variety of tasks in many IoT applications. Therefore, they require excellent specifications such as high computation processing power, large storage and memory. Further required is large amounts of storage in the cloud to conduct processing and store the data; thus, it has a high cost due to use of large resources. TinyML processes data locally in microcontroller devices that have low cost and can apply AI locally on the device itself to process the data with high performance [23]. Microcontrollers have low cost compared to other solutions due to their limited use of resources, as processor compute in the range of 1 MHz to 400 MHz. The memory for microcontroller can be of 2 KB to 512 K, whilst the storage capacity can be 32 KB to 2 MB. The cost of a Microcontroller is a few dollars in comparison to other smart IoT devices used to process data locally using DL models.
- Latency: In TinyML, data processing is executed locally in the device since the computations are performed in the device. Thus, IoT devices do not suffer from latency. The real-time local processing of data in the devices leads to faster response and rapid analysis in emergency scenarios. Besides this, the burden on the cloud is reduced [24,25].
- System reliability and data security: IoT devices require communication channels to transfer stream raw data from the IoT device to the cloud. When the data is transmitted to the cloud, it is prone to transmission errors, cyber-attacks e.g., eavesdropping or man-in-the-middle issues. Thereby, the transmitted data may be compromised or lost. According to the IBM Cost of Data Breach report 2020, the average cost of a data breach is pegged at USD 3.86 million [26]. Therefore, data need to be processed locally to limit cloud traffic. TinyML can prevent these issues through performing processing of data locally in the same device. which permits it to perform fewer transmissions with aggregated or meaningless data for an attack [23,25].

**Figure 2:**
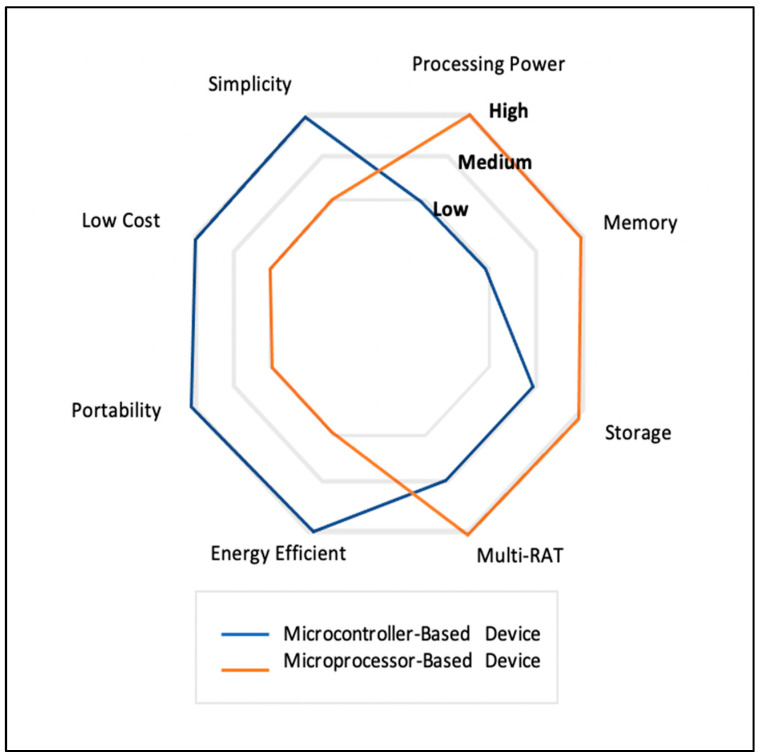
Comparison between the main characteristics of devices powered either by microcontroller or microprocessor.

Figure 2 clarifies the comparison in characteristics between two IoT devices (microprocessor and microcontroller). Microprocessors are most used in IoT with DL and have high requirements for resources such as processing power, memory, and storage. However, it is high in cost, consumes lots of memory and is not easily portable. In contrast microcontrollers are used in TinyML which have a low level of resource requirements such as processing power, memory, and storage. However, it also has low cost, energy efficiency, portability and simplicity.

## 3. Research Methodology

This section presents the methodology to locating, selecting, and critically assessing papers that address all topics related to TinyML. The objective of the paper was to import all the papers related to emerging topic which are TinyML and impart details regarding the reviewing of the research works performed in this domain and their key contributions, along with mentioning details for datasets, models, and devices. This paper was carried out following the guidelines in PRISMA (Preferred Reporting Items for Systematic Reviews and Meta-Analyses) [27]. The method is described in the following subsections.

### 3.1. Core Questions

The selection of the studies based on the whether or not the study answering research questions, the research questions comprising the focus in this study are:

1. What are the datasets, models and devices used in TinyML?
2. What are the types of application domains that are used with TinyML?
3. What are the frameworks and libraries used to develop TinyML?
4. What are the existing limitations for developing TinyML and objects for future research?

### 3.2. Search Strategy

The web-based resources (Google Scholar) and Clarivate’s Web of Science (WoS), which is a powerful and trusted database, contain various academic resources which are base databases for searching about TinyML, in addition to other databases as complementary sources: Science Direct, IEEE, MDPI and Springe. The first publication for TinyML was on 13 December 2019. The keywords used for searching are listed in Table 1.

### 3.3. Eligibility Criteria

The published studies with dataset, models and the type of devices were included. The published studies must include the experiment with outcomes described.

### 3.4. Inclusion and Exclusion Criteria

The Inclusion Criteria were select studies that aimed to: apply TinyML in various cases, develop models, use various types of datasets in TinyML, apply TinyML on different devices and develop frameworks and libraries.

Exclusion criteria included studies relating to challenges and directions for TinyML, compression model techniques as quantization and pruning studies, studies concerning memory reduction, TinyML services, technical report articles and TinyML experiments published on websites.

### 3.5. Data Extraction and Synthesis

After selecting a study for review, next an in-depth study of each selected paper was carried out. To extract the main contribution, implementation including datasets, models, devices, and the evaluation criteria for models and devices results of each paper were examined. The purpose of the data synthesis approach is to answer research questions. The information extracted from studies is described in Section 6.

### 3.6. Data Selection

**Figure 3:**
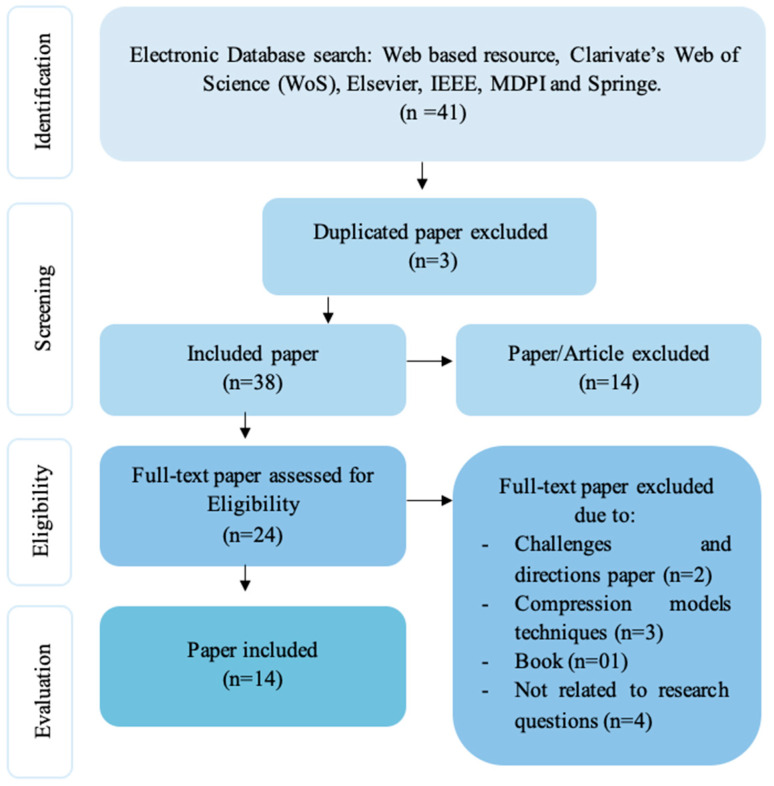
PRISMA flowchart for the study.

A total of 38 relevant published papers/articles were retrieved using search engines from the electronic data source. The first published paper on the emerging topic was on 19 December 2019, using Mendeley Desktop software to import papers and filtering a duplication. The papers were included and excluded in this study according to the preferred reporting items for systematic reviews and meta-analyses (PRISMA) statement as shown in Figure 3, which describes the process for selecting papers.

## 4. Related Work to TinyML

A methodical search was performed through the TinyML literature. We found studies concerning optimization in two aspects which are currently being carried out: optimizing models to fit into devices and optimization of framework, library, or tools. Therefore, we divided studies into two categories: The first category is focused on the studies that are concerned with the development of DL algorithms and their methodologies. Besides this, they are applied in a variety of IoT applications. There are many studies focused on developing models to fit into devices with high accuracy and high computational capabilities on various applications such as hand gestures, recognition of sign language, identification of medical masks on persons, etc. The second category is focused on the studies that are more related to design frameworks and libraries. The aim is to optimize the integration process and inference of the model into edge devices, for instance, the proposed framework specially for deploying tiny models into tiny edge devices in order to easily deploy code and inference into devices. Further projects include designing a library for quantizing the neural network for deployment on the microcontroller, convolution kernel with multiple bit precision 8, 4, and 2 bits, etc.

### 4.1. TinyML Use Cases Studies

This section presents use cases for tinyML studies. TinyML can be applied to several cases in a variety of fields. The list of use cases can include, but are not limited to, the following: sign language detection, handwriting recognition, medical face mask detection, gesture recognition, speech recognition and autonomous mini vehicles.

#### 4.1.1. Environment

The authors [5] developed an Edge Learning Machine (ELM) framework to execute ML inference in edge devices such as Microcontroller. Four algorithms were implemented on six devices from ARM Cortex-M microcontrollers released by STM-32, namely (F091RC, F303RE, F401RE, F746ZG, H743ZI2, and L452RE). Three supervised algorithms were used, which are K-Nearest Neighbor (KNN), Support Vector Machine (SVM), and Decision Tree (DT) algorithms and one unsupervised algorithm, which is an Artificial Neural Network (ANN). the performance of selected edge devices was evaluated, all algorithms and a six-dataset on each one of the devices were applied. The six-benchmark dataset is representative of IoT applications, namely Heart, Virus, Sonar, Peugeot 207 * EnviroCar, and AQI. They used the accuracy or coefficient of determination metrics to evaluate the binary and Multiclass dataset and R2 scores for the regression dataset. As a rough summary result, the best performance was that of ANN in all datasets except Sonar dataset. In contrast the SVM achieved the best results on the Heart and EnviroCar dataset. The performance of DT was on Virus and Peugeot 14. However, KNN achieved the best result in the Sonar dataset only.

#### 4.1.2. Sign Language Detection

TinyML can accommodate the sign language detection use case. A DL model was introduced to detect Sign Language Alphabet on tiny edge devices, with an aim to enable deaf-mute to easily communicate with the community. Authors in [28] proposed a model to detect the American Sign language (ASL) Alphabet and transcribed to text and speech in real time on tiny wearable IoT devices. The device has the smallest and cheapest microcontrollers. Four datasets were used that contain sign language alphabet images since the third dataset was created by authors through OpenMV H7 camera. The proposed CNN model with two augmentation techniques has been used. First, basic augmentation techniques as rotation, flip, etc. were used; second, five interpolation strategies were used in OpenCV (INTER_NEAREST, INTER_LINEAR, INTER_AREA, INTER_CUBIC, and INTER_LANCZOS4). The outcomes were 98.53% and 99.02% accuracy. The author used TF Lite to quantize the model from 32float into int8, with an attempt to deploy on OpenMV H7 board-based microcontroller STM32H743VI, which has low memory. The outcomes after deploying the model on the device showed a model with five interpolation strategies outperforming another model with 98.84% accuracy and 20 frames per second (FPS), whilst the accuracy of the model with basic augmentation techniques was 95.24%.

#### 4.1.3. Handwriting Recognition

TinyML can help to easily train and deploy the model to recognize handwriting on a microcontroller and has the low performance to pave the way to the Internet of Conscious Things. In [25], various models are developed to apply to TinyML. They conducted an experiment on the MNIST dataset using the STMicroelectronics NUCLEO-F746ZG board through the X-CUBE-AI tool. For the training phase, the Neural Network (NN) algorithm has been implemented with two hidden layers between input and output using the TensorFlow library and then built CNN using Keras library. CNN improved the training accuracy from 97.25% to 99% with ReLu and Sigmoid activation functions. The weights of the NN and Keras models were around 15 MB and 7172 KB, respectively. The Keras model was sequentially deployed on the real embedded devices by converting the model using TF Lite and TFliteConverter tools. Since TFLite and TFliteConverter tools reduce the model size to 2.4 MB, embedded devices have memories of a few hundred KB. Pruning and post-training Quantization techniques were applied with a slight loss in accuracy; pruning enhances the speed of inference of the model and reduces the amount of energy required. Post-training quantization minimizes energy consumption and computing power demand. The result of the prediction model was 100% accuracy, while the model consumed 135.68 KB of RAM, 668.97 KB of flash memory, and 330 ms computation.

#### 4.1.4. Medical Face Mask Detection

COVID-19 has suddenly put the world in a cautious mode where cough-related medical face mask detection has become an important task. TinyML can help IoT-based smart health to be more secure and preserve battery. Puranjay Mohan et al. [29] presented a tiny CNN model to detect Medical Face Masks using real-time on devices. This was carried out considering the preservation of reliability and privacy without transmitting images to the server and the results being sent back to the application. Four datasets have been used with various augmentation techniques to train and test the model. Notably, the third dataset was constructed by the author using an OpenMV camera to find the classification metrics. A CNN model was proposed for use on the OpenMV Cam H7 housing STMicroelectronics’ STM32H743VI. In addition, a SqueezeNet model was modified in order to compare the results with the proposed model. Overall, after performing the quantization technique on all three models to reduce the size, the results demonstrated that only the proposed model can deploy on the device, with 138 KB model size; the inference speed was 30 FPS and the accuracy was 99.83%.

#### 4.1.5. Gesture Recognition

Gesture recognition is considered a promising field for the TinyML domain. Several methods are applied to hand and foot gestures using the limited resources of IoT devices. A study in [30] tries to deploy DL on compact wearable devices for gesture recognition that in turn helps to reduce the consumption of both power and time. They created a prototype ring sensor to be used in collecting data and to deploy NN models on it. The ring sensor consists of Taiyo Yuden EYSHSNZWZ NRF52 as the main controller, Bluetooth modem and an ST Micro LIS3DH three-axis acceleration data. They collected data for ten gestures (from 0 to 9) from the device to train the Keras model and a multilayer long short-term memory (LSTM) model in TensorFlow. The result showed that the Keras model did not perform well, with 19% accuracy, while LSTM attained 93% accuracy in the training phase, 83% validation accuracy, and 84.5% accuracy in the final evaluation. The model size for LSTM was 2.8 MB. Unfortunately, they did not deploy an LSTM model on the device due to the lack of support for it by TF Lite Micro. Other authors have recognized and classified a set of hand gestures using classified time series values.

In [31], an ANN model was developed to recognize and classify the time series values of a sensor for the set of hand gestures (Forward, Backward, Select, and Abort). They used a hand gesture sensor that has a compound eye camera with an ATMega4809 microcontroller to implement the experiment. The microcontroller characteristics were 6 KB of RAM, 48 KB of flash memory and a clock speed processing up to 20 M. They created a dataset by recording various gestures performed by an entire hand, an arm or fingers from different distances, then performing synthetic data as in Table 2. Two models are used: RNN and Feed Forward Neural Networks (FFNN). The performance of the models showed that the FFNN outperform the RNN. FFNN achieved 84% accuracy with the ReLu activation function, while RNN achieved 83% accuracy. Both algorithms confirmed that 2 KB RAM and 32 KB flash memory of the ATMega328P microcontroller are sufficient to enable a 40 Hz frame rate.

TinyML is also applied to recognition of foot gestures. The authors in [32] proposed a low-cost wearable system that has the ability to recognize foot gestures. Furthermore, it can transfer messages via long-range (low-power wide-area networks) using LoRa radio technology in an emergency case. Once the person is exposed to danger, the system notified his/her emergency contacts using secret methods. The proposed classifier is based on the Neural Network to differentiate between activities (such as walking, jogging, and standing) and two gestures (double-tap at the toe tip and double-tap at the heel). The proposed system consists of two force sensors placed at the top and heel of the shoe and connected to LPWAN MCU (containing ESP32 MCU and an RFM95 LoRa modem). They also used another LPWAN as a receiver that is directly connected to the laptop as a gateway and Arduino-LMIC software. They collected 30,000 data points for ten minutes, then divided them into batches of 100 samples (2 s of recording); a total of 300 arrays for each activity/gesture were created. The experimental was implemented using the NN model indoors and outdoors via two people. The overall accuracy result for indoor tests was 99.33%, whereas outdoor tests showed a slight drop in accuracy, with 97.5%.

#### 4.1.6. Speech Recognition

Speech recognition is a popular application for IoT with DL. TinyML is applied to speech recognition to solve challenges of speech recognition on tiny edge devices such as latency, computing resources and memory constraints. Study [33] presented TinySpeech for speech recognition on tiny devices. TinySpeech aims to build a deep convolution network that has low architecture, low computation on the devices and required low storage. First, they used the Google Speech Commands dataset for recognition of limited-vocabulary speech to train the model. Next, they applied pre-processing to the dataset to extract the Mel-frequency cepstral coefficient (MFCC) to feed into the TinySpeech network. The design of the TinySpeech network consists of attention condensers and machine-driven design exploration. The new attention condenser was incorporated into generative synthesis. During the generative process, they take the four constructed generators, namely TinySpeech-X, TinySpeech-Y, TinySpeech-Z, and TinySpeech-M, which were generated in different stages with different levels of performance and efficiency. The results offered by TinySpeech X achieved the highest accuracy, with 96.4%. TinySpeech-Y, TinySpeech-Z and TinySpeech-M achieved 93.6%, 92.4%, and 91.9% accuracy respectively. TinySpeech-Z possesses the smallest architecture with a model size of 21.6 kbits and 92.4% accuracy. In comparison with other studies based on the Legendre Memory Unit (LMU), the model size was 49 kbits and achieved 92.7% accuracy.

#### 4.1.7. Autonomous Mini Vehicles

Autonomous mini vehicles are used in many fields such as smart industry, smart environment, smart monitoring, etc. TinyML improves the performance of autonomous mini vehicles through enhancing the learning complex action a few times with low consumption of energy. Miguel de Prado et al. [34] enable the execution of DL on low-power autonomous driving vehicles with an aim to enhance the performance, e.g., actions/s by learning complex challenges. Thus, it can take decisions (image classification) in a complex environment with less latency and low energy. They substituted a Computer Vision Algorithm (CVA) which was predicted only under stable light conditions by CNN by using LeNet5 model. Subsequently, modified the LetNet5 and constructed a family of networks called Vehicle Neural Networks (VNNs). They created three datasets (Dset-2.0, Dset-1.5 and Dset-1.0), each one containing 1000 per class in the training set and 300 for the test set; details of the datasets. After training the VNNs on the combined datasets (Dset-All), post-training quantization was applied for both weights and activations on the VNN model to fixed-point 8-bit to reduce memory and power consumption. The authors deploy VNN networks on different platforms and devices such as GAP8 (GAP8, a parallel ultra-low-power RISC-V SoC), STM32L4 (Cortex-M4), and NXP k64f (Cortex-M4). The results obtained VNN3, VNN4, and LeNet5 models reach an accuracy of 98.74% on the reinforced dataset Dset-All (I1, I2, and I3) by using GAP8.

### 4.2. Studies Related to Design TinyML Frameworks and Libraries

This section offers studies related to the design environment as frameworks (e.g., TensorFlow Lite Micro) and libraries. These aim to address issues encountered by developers using code in devices that have restricted resources while maintaining successful performance, e.g., high accuracy and low inference time. In addition, we present studies that optimize the power consumption and memory of devices by using design libraries and enabling efficient learning in the devices. Subsequently, various models are implemented into different devices to validate them.

#### 4.2.1. TinyML Framework Studies

TinyML frameworks have been developed by some developers, research groups, and incorporations. Robert David et al. [35] demonstrate use of the TensorFlow Lite Micro (TF Micro) framework to tackle issues that the developers faced when developing and training models, and after that, deploy them on tiny devices. TF Lite Micro introduced many benefits for developers and hardware vendors, such as providing unified platforms with various features. It allows mitigation of the cost for training and deploying models and further allow vendors to optimize incrementally their kernel. On the other hand, the system has undergone many tests using different datasets and devices. They used two devices; the first was the Sparkfun Edge, which has an Ambiq Apollo3 MCU. The second device, Xtensa Hifi Mini, has digital signal processors (DSP), which were based on the Cadence Tensilica architecture (Cadence, 2020) using two benchmark models which are the VWW model and the Google Hotword model. Overall, the results show that the optimized models achieved high performance specifically in total run time and memory. The results of the VWW model were 4.857 K and Google Hotword achieved 36.4 K on the Sparkfun Edge device. VWW achieved 49,952.3 K and Google Hotword 88.4 K on Xtensa HiFi Mini DSP. Our benchmarks are INT8 TF Lite models in serialized FlatBuffer format.

Another framework was designed by authors in [17], who proposed the MCUNet framework which combined the designs of TinyNAS and TinyEngine simultaneously into an optimized inference of ML on MCU. TinyNAS is an efficient two-stage piece of neural architecture, whereas the TinyEngine is a lightweight inference engine. They used multiple benchmark datasets, namely ImageNET and Wake Word; Visual Wake Words (VWW) and Google Speech Commands (GSC). They used many devices such as the STM32F746 and STM32H743. Quantization techniques were applied to convert the model into an 8-bit version. The results of the co-design TinyNAS with TinyEngine on the ImageNET dataset and STM32F746 device achieved the best accuracy, with 62%. MCUNET achieved the highest accuracy on the STM32H743 device, with 70.0%. Meanwhile, MCUNET outperforms other models for SRAM, with 0.49 MB, and consumed 0.9 MB of Flash memory. Furthermore, MCUNET improves the performance of different latency constraints with 49.9% at 5 FPS and 40.5% at 10 FPS. Concerning the results of the VWW and GSC dataset, MCUNET achieved state-of-the-art accuracy on visual & audio wake words tasks and runs 2.4–3.4× faster than MobileNetV2 and ProxylessNAS-based solutions with 3.7–4.1× smaller peak SRAM.

Several authors have tried to deploy DL into different tiny devices in order to predict with high efficiency. However, there are challenges due to a lack of a standard framework to implement TinyML in devices from different companies. The authors in [6] proposed a general environment for deploying DL model into various tiny devices. The environment consists of TF Lite and Mbed OS software with an aim to enable inference of DL in many edge devices from different companies, such as ARM and NXP. In addition, a comparison was conducted to evaluate the performances of the edge device and a personal computer (PC) using the family of MobileNet (V1, V2, and V3) on a single case of human detection. VWW was used to evaluate the model, since the dataset was split into 115,287 for training the model and 100 images (50 images containing a person and 50 images that did not contain a person) for testing. The authors experimented on both STM32H747I-Disco and OpenMV Cam H7 and divided them into three phases. The first phase aims to ensure the proposed environment can integrate DL into devices. The result was that the MobileNet-V2 achieved high accuracy on STM, with 88%, and high latency, with 220 ms. The second phase of the experiment aims to evaluate and compare the performance of different models on edge devices and PC. The results of the comparison showed the MobileNet-V2 model with (Depth Multiplier: 0.1 and Resolution: 96 × 96) on STM and PC attained a high accuracy of 88%, with the highest model consumed for (Matrix size: 213,184 KB and RAM: 138,240 KB). The experiment had three aims to analyze the effect of application during and after quantization on MobileNet-V2 using STM. The result showed that quantization after the training was better to improve the accuracy model.

#### 4.2.2. TinyML Libraries Studies

TinyML Designing libraries optimize power and memory consumption in tinyML and enable efficient learning in devices. The authors implement DL models into different devices with constraint resources to validate them.

A study in [36] also attempted to solve issues in memory constraints on the microcontroller. They presented an open-source CMix-NN library which is a Mixed Low-Precision library that aims to quantize the neural network to deploy on microcontrollers. CMix-NN provides a convolution kernel with multiple-bit precision 8, 4 and 2 bits (independent tensors, quantization of weight, and activations in 8, 4, and 2 bits). To evaluate the system, the ImageNet dataset and MobileNet-V1 model were used for deployment on an STM32H743 SoC device for the classification task. The performance of CMIX-NN was more accurate than other libraries such as X-CUBE-AI (FloatPoint 32) and CMSIS-NN (8-bit), achieving the highest accuracy on the ImageNet problem, with 68%. However, the model size was 1.97 MB, which is larger than the others, the latency was 1.86 s and the energy was 491.15 μJ.

On the other hand, a novel method can be provided to reduce the memory size of the training model to fit into the tight-memory constraint of the device, with an aim to enable efficient learning on the device. The study in [37], introduced a Tiny-Transfer-Learning (TinyTL) model, which aims to reduce the model size by freezing the weight of the pre-trained feature extractor with a continuous update and learning a bias module. To preserve the adaption capacity, a lite residual module is presented, which in turn polishes the feature extractor through learning small residual feature maps. The authors conducted extensive experiments to evaluate the proposed methods through performing a comparison with other transfer learning. The comparison was conducted on nine image classification datasets, namely Flower, Cars, CUB, Food, Pets, Aircraft, CIFAR10, CIFAR100, and CelebA, as in Table 3. In the experiment, they used ProxyNAS-mobile as a pre-trained model, then applied TinyTL and the full fine-tuned network (FT-Full) to evaluate the model. The result showed that TinyTL reached the level of accuracy of FT-Full, consuming 37 MB of memory for all datasets while FT-Full consumed 391 MB. Further, with a combined feature extractor adaptation (FA) TinyTL saves 66 MB of memory, compared with the FT-Full with Inception-V3 model which consumed 850 MB; also, FA achieved the highest accuracy on the Pets dataset, with 93.5%.

## 5. Discussion and Findings

Based on the related works of tinyML, we have derived the following findings concerning models, datasets, and devices. In addition, we analyze the main results for each section. The following section is divided into three subsections: findings based on the dataset, findings based on the machine and DL model, and findings based on the devices.

### 5.1. Findings Based on the Datasets

**Figure 4:**
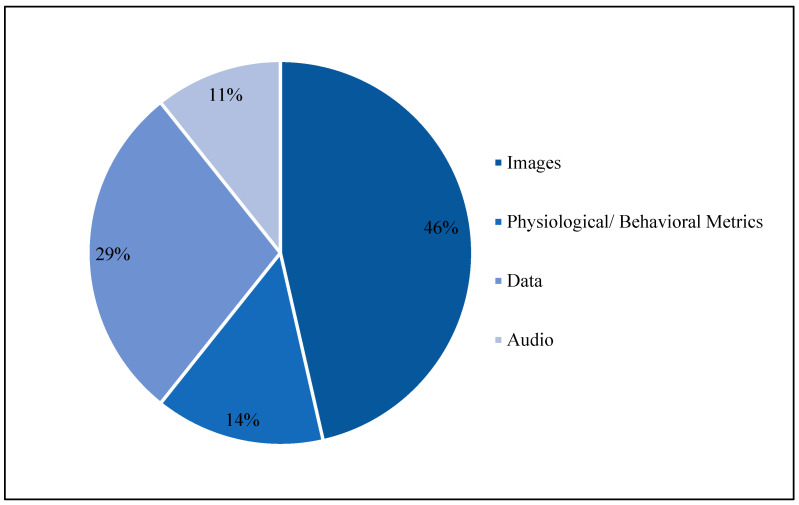
Dataset type distribution in previous studies.

There are many types of datasets used in TinyML studies, such as images, audio, physiological/ behavioral metrics and other data, e.g., virus dataset, sonar dataset, etc. Figure 4 shows the ratio of dataset types that were used. Images constitute 46% of the data. Next, other data were used, e.g., a sonar dataset that includes data for Miners and Rocks to analyze the materials. Next, physiological/behavioral metrics constitute 29%, and contain data for biometrics such as a heart dataset or activity recognition in people, such as walking, jogging and standing. Audio data constitute 11%; notably, the GSC dataset was the most used in the audio dataset.

Table 4, presents the datasets of the studies. The first section depicts image datasets; the most frequently used datasets in previous studies were the ImageNET and VWW datasets. The authors used some of the classes from these, such as Flower, CIFAR10, CIFAR-100, etc., for training the models. The second section comprised physiological/behavioral metrics, including various datasets such as heart, hand gesture, foot, and activities gestures dataset. Physiological/behavioral scale datasets consist of time-series data recorded by smart sensors for recognition. The third section contains the datasets related to many fields as viruses and transportation; these contain data to predict the surface of the road and traffic. They also contain datasets such as Dset-2.0, Dset-1.5 and Dset-1.0 that have data created by authors related to autonomous vehicles. The final section is audio; we found only one dataset used in studies created by Google, who designed a dataset for tiny devices called Google Speech Commands (GSC) dataset. GSC includes Speech Commands data for keyword spotting (e.g., “Hey Siri”), requiring classifying a spoken word from a vocabulary of size 35.

The details of each dataset are mentioned in Table 5. The description of each dataset was used in the studies and, in addition to the total number of datasets the number of training and testing datasets was also obtained, since 80% of samples were employed for training and the other 20% for testing. Many studies used the augmentation techniques through training tiny models to increase the number of samples in the dataset, e.g., basic augmentation techniques and OpenCV’s interpolation augmentation in order to obtain accurate results and high performance.

### 5.2. Findings Based on the Machine and Deep Learning Models

**Figure 5:**
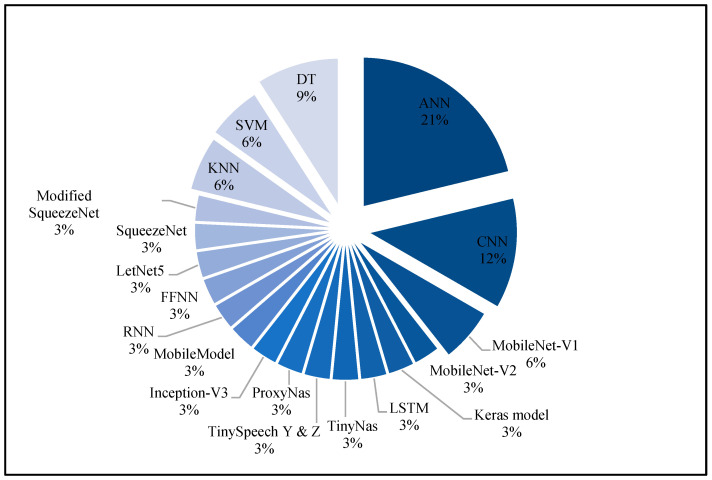
Machine and deep-learning models distributed in previous studies.

Figure 5 shows model types that achieved the best results in previous TinyML studies. The results obtained show that the most common models used were ANN with 21%, CNN with 12% and MobileNet-V1 with 6%. Despite this, neural networks (NN) were the dominant force in traditional ML as well as achieving the best result in TinyML. On the other hand, ML algorithms that are non-NN based, such as Decision Tree (DT) represent 9%, Support Vector Machine (SVM) with 6% and k-nearest neighbors (KNN) with 6% achieved the best results in some cases for TinyML that have data using tiny devices.

Recently, ML has specifically enabled DL on tiny devices which have constrained resources; low-computing and low-memory devices are feasible. However, we explored challenges that prevent the community from producing and developing models and enhancing accuracy. For example, one problem is a lack of a standard methodology for deploying models on all devices. Most companies producing devices have their own framework. In addition, no common models are used, such as MobileNet models for mobile devices. Thirdly, for compression techniques, there are no standard methods to DL, where the performance of compression is shown once the model has been deployed on the device.

### 5.3. Findings Based on the Devices

**Figure 6:**
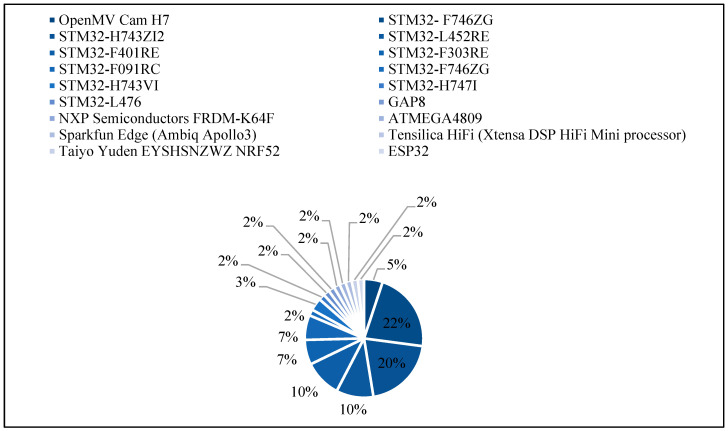
TinyML devices distribution in previous studies.

In this section, the devices that were used in tinyML studies as shown in Figure 6 that demonstrate the percentage of device usage are examined. Furthermore, in Table 6, the characteristics of each device are illustrated. We notice that the costs of these devices are low, ranging approximately from USD 16.00 to USD 28.00, due to the use of limited resources. In addition, we notice that most of the devices make use of the STM32-microcontroller. STM32 is a family of 32-bit microcontroller-integrated circuits by STMicroelectronics [61]. STM32-F746ZG and STM32-H743ZI2 devices were both frequently used in studies too, with 22% and 20% frequency. These devices achieved high performance and successful inference of DL models. STM32-F746ZG has 1 MB of flash memory and has 340 KB of RAM, whereas STM32-H743ZI2 has 1 MB of flash memory and 2 MB of RAM. These devices have costs of USD 24.47 and USD 27.00, respectively.

We found that the devices used mostly achieved high performance as well as having many other advantages. First, it has a Nucleo-144 board that presents an affordable and flexible way for the users to create a creative application and build prototypes. Second, it works on the STM32 Cube.AI toolkit, which is a free software library and publicly available. STM32 Cube.AI toolkit permits integration of pre-trained Neural Network models within STM32 ARM Cortex- M-based microcontroller, as well as generate suitable C code from Neural Network models by Keras, TensorFlow, Lite Caffe and other frameworks. STM32 Cube.AI toolkit allows storage weights and activation buffers of large Neural Networks in flash memory and RAM, respectively. In addition, it integrates with the ST-LINK debugger/programmer; thus, it does not require any separate probe.

## 6. TinyML Limitation

TinyML achieved remarkable breakthroughs in many cases of use. In addition, high accuracy was obtained with various DL models on IoT devices with limited resources. However, despite the powerful perception capability of TinyML it has many limitations that constitute obstacles to the development of TinyML. Some of TinyML’s limitations are presented in the following subsections.

### 6.1. Device Heterogeneity

Heterogeneity of hardware and software presents the main challenge to adoption of the TinyML system. Authors have tried to provide various methods to reduce the size of ML and use it in devices with low resources without a drop in their accuracy. However, the result of accuracy is not the same as on desktop computers. Due to the fact that manufacturing companies’ embedded systems do not have a generalized method, each company has its own software to fit the system into their devices. Thus, there is no standardized method including software as framework to implement and execute DL models inside various devices from different companies without drops in accuracy. Developing a generalized framework is necessary for increasing adoption and awareness of TinyML [62,63].

### 6.2. Process Power

MCU from various company such as ARM Cortex have high-end processors, but their processing power and speed is still low in comparison with cloud-based systems as edge devices. Cloud based systems have the ability to process massive amounts of data with large DL models. Therefore, perhaps quality of data analysis will be hampered when shifting from cloud to device.

### 6.3. Limited Memory

Limited memory with only a few KB is one of the main motivations for the creation of TinyML. Traditional inference of DL models requires drastically higher peak memory (in the order of gigabytes) than TinyML devices can provide. This poses challenge, therefore creating a need for innovation in optimization techniques appropriate for each algorithm. As a consequence of compression DL models, it is important that the performance of parameters matches the original model [64].

### 6.4. Limited DL Models

There is lack of development DL models that achieve good performance with high accuracy and small models. Still, DL models process a variety of data with huge parameters and accrue results. Thus, TinyML needs to optimize the training and inference DL models to analyze data on low-power devices without loss of accuracy and with low latency.

## 7. Conclusions

TinyML is a dynamic and rapidly developing field that requires interoperability between IoT devices to ensure stability and continuous progress. TinyML is a coming field that intersects hardware, software, and machine-learning algorithms and that is gaining enormous traction. Recent studies in this field contain building DL networks with sizes of a few hundred KBs. TinyML is deployed into tiny devices to turn them into intelligent devices. TinyML has many benefits, e.g., reduced computation, power consumption and response time. In this paper, we present an overview of TinyML and showed its advantages. After that, we reviewed two kinds of TinyML studies; first, some studies developed models and applied TinyML in different IoT applications using a variety of devices with limited resources. Second, other studies have developed libraries, frameworks for optimization using TinyML. Subsequently, analysis and discussion of the main findings of TinyML studies were presented, including datasets, models, and devices to draw the main findings of TinyML overall. Lastly, we discuss the main limitations that constitute obstacles to the development of TinyML applications, which will guide researchers to solve open problems in the TinyML field for future works.

## Figures and Tables

**Table 1:** Search plan/approach.

Source	Criteria
Database	web-based resources and Web of Science
Date of publication	2019–2021
Keywords	TinyML Tiny Machine Learning Tiny Deep Learning Deep learning AND Ultra-power-device Deep Learning AND Microcontroller
Language	English
Type of publication	Conference Proceedings Research Article ArXiv preprint
Inclusion criteria	TinyML Use Cases paper Develop Framework Develop library for TinyML
Exclusion criteria	Challenges and directions paper Compression models techniques on Ultra power devices Not related to research questions

**Table 2:** Comparison between earlier studies related to DL methodologies based on the used models, the results, and the inference devices.

Study	Model	Model Result in Desktop			Inference in Devices			Result after Deployment		
		ACC	Model Size	Platform	Name	Platform	Metrics	Latency	Ram	Flash Memory
[5]	SVM	84%	-	-	All devices	STM X-Cube-AI expansion package, and C language platform	All 84%	<1 ms	-	-
	ANN1	99%—<1 m	-	-	F746ZG		Both 99%	1 ms	-	-
					H743ZI2					
	KNN	99%—<1 ms	-	-	F746ZG		Both 92%	Both 10 ms	-	-
					H743ZI2					
	ANN2	99%—<1 ms	-	-	F746ZG		Both 99%	Both <1 ms	-	-
					H743ZI2					
	DT	99%—<1 ms	-	-	F746ZG		Both 99%	Both <1 ms	-	-
					H743ZI2					
	ANN3	0.86	-	-	F401RE		0.86 R2	<1 ms	-	-
					F746ZG					
					H743ZI2					
					L452RE					
[25]	NN CNN	97.25% 99%	15 MB 7172 KB	TFLite and TFliteConver	F746ZG	X-CUBE-AI tool	100%	330 ms	135.68	668.97
[28]	CNN1 CNN2	98.53% 99.02%	185 KB	TF Lite	OpenMV H7 board STM32H743VI.	TF-Convert	95.28% 98.84%	20 FPS	-	-
[29]	CNN	99.83%	1.5 MB	-	OpenMV H7 STM32H743VI.	-	99.83%	30 FPS	-	-
	SqueezeNet	98.50%	8.0 MB				98.53%			
	SqueezeNet2	98.93%	3.8 MB				98.99%			
[30]	Keras	19%	-	TensorFlow	Taiyo Yuden EYSHSNZWZ NRF52	-	-	-	-	-
	LSTM	93%	2.8 MB	TensorFlow	-	Tensor Flow Lite Micro- Not Support it	-	-	-	-
[31]	RNN FFNN	61%	-	-	ATMega4809	TensorFlow.	84%	Both 40 Hz	Both 2 KB	Both 32 KB
							93%			
[32]	NN	-	-	-	ESP32	Arduino-LMIC software	99.33% indoor 97.5%. outdoor	2 min per activity. 0.5 per gesture.	-	-
[33]	TinySpeech-X.	96.4%	-	TensorFlow Lite for Microcontroller	-	-	-	-	-	-
	TinySpeech-Y	93.6%	48.8 KB							
	TinySpeech-Z	92.4%	21.6 KB							
	TinySpeech-M	91.9%	-							
[34]	LetNet5 model Vehicle Neural Networks (VNN1,2)	99.53% 79.62% 81.27%	-	PyTorch	STM32 L476 board	X-Cube-AI (float32 operations)	-	14.15 ms	80 MHz	-
					NXP k64f	ARM CMSIS-NN.	-	0.97	120 MHz	-
					GAP8	PULP-NN	-	1000 fps with 1 ms	-	-

**Table 3:** Comparison between earlier studies related to Design tinyML frameworks and libraries based on the used models, the results and the inference devices.

Study	Model	Model Result in Desktop			Inference in Devices			Result after Deployment		
		ACC	Model Size	Platform	Name	Platform	Metrics	Latency	Ram	Flash Memory
[37]	VWW model Google Hotword Optimized	-	-	-	Sparkfun Edge Xtensa Hifi Mini digital signal processors (DSP)	TensorFlow Lite Micro	-	-	4.857 KB 49.95 KB 36.4 KB 88.4 KB	81.79 KB 12.80 KB
[17]	TinyNAS model and TinyEngin library	-	-	-	STM32F746	MCUNET	61.8%	49.5% at 5 FPS and 40.5% at 10 FPS	0.49 MB	1.9 MB
							87%	89% at 5 FPS and 87% at 10 FPS	91 KB	<140 MB
							-	94% at 5 FPS and 91% at 10 FPS	-	<124 MB
									36.4 KB 88.4 KB	
[6]	MobileNet-V2	-	-	-	STM32H747I-Disco	TensorFlow Lite and MbedOS	88%	220 ms	138,240 KB	Matrix Size: 611,912
					OpenMV Cam H7					
[36]	MobileNet-V1	-	1.97 MB		STM32H743 SoC	CMix-NN	68.2%	1.86 s	-	-
[35]	ProxyNAS with FT-Full	-	-	Computation theory	-	-	-	-	391 MB	-
	ProxyNAS with tinyTL								65 MB	
	Inception-V3 with FT-Full								850 MB	
	TinyTL with FA								66 MB	

**Table 4:** Summary of datasets used in TinyML studies including input type.

Input Type	Dataset	Reference
Images	Handwritten digits	[38]
	Sign MNIST dataset from Kaggle	[39]
	Kaggle ASL dataset (26 classes)	[40]
	ASL dataset created by authors	[28]
	Kaggle ASL Alphabet test set.	[41]
	Face Mask 12 K Images dataset from Kaggle	[42]
	Face Mask Classification dataset from Kaggle	[43]
	Face Mask Dataset created by authors	[29]
	Face Mask testing dataset created by authors	[29]
	ImageNET (Flower, CUP, Pets, Food, CIFAR10 and CIFAR100)	[44,45,46,47,48,49,50,51,52]
	Visual Wake Word (VWW)	
Physiological/ Behavioral Metrics	Heart dataset	[53]
	Hand gesture recorded ((Forward, Backward, Select and Abort) using fingers created by authors	[30]
	Hand Gesture data (0–9) Created by the authors	[31]
	Foot gesture and activity data Created by authors	[32]
Data	Virus dataset	[54]
	Sonar dataset	[55,56]
	Peugeot 14	[57]
	Peugeot 15	[57]
	EnviroCar	[58]
	Air Quality Index (AQI)	[59]
	Dset-2.0 created by authors	[34]
	Dset-1.5 created by authors	[34]
	Dset-1.0 created by authors	[34]
Audio	Google Speech Commands (GSC)	[60]

**Table 5:** Summary of dataset descriptions used in previous TinyML-based studies.

Study	Dataset	Description	Total	Training Dataset	Testing Dataset
[5]	Heart dataset	Heart dataset produced by the University of California Irvine (UCI), contains 13 features. In these, 0 represents an absence of coronary heart disease (CHD) in the patient and labels 1–4 represent the presence of CHD	300 data	-	-
	Virus dataset	Developed to be used in data traffic analysis	-	-	-
	Sonar dataset	Contains reading sonar system for two classes (Miners and Rocks) to materials analysis	208 data	-	-
	Peugeot 14	Contains different parameters from cars to predict road surface	8615 data	-	-
	Peugeot 15	Contains different parameters from cars to predict the traffic	8615 data	-	-
	EnviroCar	Contains anonymized tracks of car measurements collected by citizen bus The used dataset contains approximately 1.7 million data points. Each data record contains 24 attributes reflecting sensor values of the vehicle (e.g., speed, rpm...)	Around 1.7 million data point	-	-
	AQI	Air Quality Index (AQI) dataset includes measurement of air quality for one year in Australia.	Real-time data from website	-	-
[25]	Handwritten Digits	Handwritten digit images from (0 to 9)	70,000 images	60,000	10,000
[28]	Sign MNIST dataset	Used 24 of the 26 letters of the alphabet in English, leaving out the letters J and Z	34,627 images	27,455 images	7172 images
	Kaggle ASL dataset	Used 3000 images per class, 24 classes for 26 letters of the alphabet in English, except for J and Z	72,024 images	72,000 images	24 images
	Sign Language dataset	Used 400 images for each of the 24 classes, used inter_area interpolation OpenCV to downscale the images into 28 × 28	400 images	40 images	360 images
	Kaggle ASL Alphabet test set.	30 images per 24 classes, used as generalization dataset or as final testset	720 images	-	-
[29]	Face Mask 12 K Images Dataset	Faces images with/without a mask with a variety of backgrounds and cropped to face region	58,960 images.	58,960 images	-
	Face Mask Classification dataset	Face images with/Without Mask	22,200 images	22,200 images	-
	Medical face OpenMV	Used the OpenMV Cam H7 camera to create a dataset. The size of images was 200 × 200 then saved on the SD Card of the development board	49,895 images	49,895 images	-
	Medical face testing dataset OpenMV Dataset.	Used OpenMV camera to create a dataset	4794 images	4794 images	-
[30]	Hand Gesture data from numbers (0–9)	Data for 10 numbers of gestures (from 0 to 9)	1000 gestures	-	-
[32]	Foot gestures and activity data	Set of data activities (walking, jogging, standing) and two gestures (double tap at the toe tip and double tap at heel).	30,000 data	24,000 data	6000 data
[31]	Hand gestures	A set of gestures were recorded such (Forward, Backward, Select and Abort) using fingers, arms and entire hands.	-	-RNN 15 data recorded then augmented to 540 -FFNN 350 data recorded then augmented to 1400	24 signals
[33]	Google Speech Commands (GSC) dataset.	65,000 of 1 s verbal command for short words with background noise.	65,000	-	-
[34]	Dset-2.0	Dset-2.0 contains samples (clear images) with (2.0 ms)	-	1000 sample	300 sample
	Dset-1.5	Dset-1.5 contains samples with (low-contrast images) with (1.5 ms)	-	1000 sample	300 sample
	Dset-1.0	Dset-1.0 contains samples (low-contrast images) with (1.0 ms)	-	1000 sample	300 sample
[17]	ImageNET:	the standard large-scale benchmark for image classification consists of Set of 1000 object categories containing internal and leaf nodes, but do not interfere with each other.	10,000 images	-	-
	Wake word: Visual Wake Word (VWW):	VWW: is a set of natural images of a complex day. Each image classifies to label 1 images present (Person) or 0 (Not Person)	5000 images	-	-
	Wake word: Google Speech Commands (GSC) dataset	GSC: Speech Commands is an audio dataset for keyword spotting (e.g., “Hey Siri”), requiring classifying a spoken word from a vocabulary of size 35.	-	-	-
[36]	ImageNET Dataset	Set of 1000 object categories contains internal and leaf nodes.	200,000 images	50,000 images	150,000 images
[6]	VWW	VWW: is a set of natural images of a complex day. Each image classifies to label 1 images present (Person) or 0 (Not Person)	115,387	115,287 images	100 images
[35]	9 datasets (Flower, Cars, CUB, food, Pets, Aircraft, CIFAR10, CIFAR-100 and CelebA)	Used ImageNet at pre-train on eight object classification datasets (Flower, Cars, CUB, Food, Pets, Aircraft, CIFAR10 and CIFAR-100) used VGG Face2 as a pre-train to one human facial attribute classification dataset (CelebA)	-	-	-
[37]	Visual Wake Word (VWW)	Each image classifies to label images (1 present Person or 0 Not Person)	115 k	115 k images	8 k

**Table 6:** Summary of TinyML devices used in different previous studies.

Processor	Flash Memory	RAM	Processor Speed (MHz)
STM32-L476RG	1 MB	128 KB	80 MHz
STM32-H743VI	2 MB	1 MbB	480 MHz
STM32 Nucleo-64 F091RC	256 KB	32 KB	48 (max: 48)
STM32 Nucleo-64 F303RE	512 KB	80 KB	72 (max: 72)
STM32 Nucleo-64 F401RE	512 KB	96 KB	84 (max: 84)
STM32 Nucleo-144 F746ZG	1 MB	340 KB	96 (max: 216)
STM32 Nucleo-144 H743ZI2	2 MB	1 MB	96 (max: 480)
STM32 Nucleo-64 L452RE	512 KB	160 KB	80 (max: 80)
STM32H747I-Disco_CPU (ARM Cortex M4+ ARM Cortex M7)	1 MB	2 MB	240 MHz (M4) + 480 MHz (M7)
STM32H743VI	2 MB	1 MB	400 MHz
GAP 8 based PULP architecture	512 kB	80 KB	22.65 Giga Operations Per Secon (GOPS)
NXP Semiconductors FRDM-K64F	1 MB	256 KB	120 MHz
ATMEGA4809	48 KB	6 KB	20 MHz
Arm CPU Cortex-M4	0.38 MB	1 MB	96 MHz
Xtensa DSP HiFi Mini	1 MB	1 MB	10 MHz
STM32H743 SoC- ARM Cortex- M7	2 MB	512 KB	480 MHz
Sparkfun Edge (Ambiq Apollo3), Arm CPU Cortex-M4	1 MB	0.38 MB	96 MHz
Tensilica HiFi, Xtensa DSP HiFi Mini processor	1 MB	1 MB	10 MHz
ESP32	448 KB	520 KiB SRAM	160 MHz–240 MHz
Taiyo Yuden EYSHSNZWZ NRF52	512 KB	64 KB	2402 MHz–2480 MHz
OpenMV Cam H7—Processor (ARM Cortex M7 480 MHz)	2 MB	1 MB	480 MHz
STM32H747I-Disco_CPU (ARM Cortex M4 + ARM Cortex M7)	1 MB	2 MB	240 MHz (M4) + 480 MHz (M7)
STM32H743VI	2 MB	1 MB	400 MHz

## References

[1] Hamdan S., Ayyash M., Almajali S. (2020). Edge-Computing Architectures for Internet of Things Applications: A Survey. Sensors.

[2] Wu Z., Qiu K., Zhang J. (2020). A Smart Microcontroller Architecture for the Internet of Things. Sensors.

[3] Signoretti G., Silva M., Andrade P., Silva I., Sisinni E., Ferrari P. (2021). An Evolving Tinyml Compression Algorithm for IoT Environments Based on Data Eccentricity. Sensors.

[4] Chen Y., Zheng B., Zhang Z., Wang Q., Shen C., Zhang Q. (2020). Deep Learning on Mobile and Embedded Devices: State-of-the-Art, Challenges, and Future Directions. ACM Comput. Surv..

[5] Sakr F., Bellotti F., Berta R., De Gloria A. (2020). Machine Learning on Mainstream Microcontrollers. Sensors.

[6] Gorospe J., Mulero R., Arbelaitz O., Muguerza J., Antón M.Á. (2021). A Generalization Performance Study Using Deep Learning Networks in Embedded Systems. Sensors.

[7] Atitallah S.B., Driss M., Boulila W., Ghezala H. (2020). Ben Leveraging Deep Learning and IoT big data analytics to support the smart cities development: Review and future directions. Comput. Sci. Rev..

[8] Bhattacharya S., Somayaji S.R.K., Gadekallu T.R., Alazab M., Maddikunta P.K.R. (2020). A review on deep learning for future smart cities. Internet Technol. Lett..

[9] Wang F., Zhang M., Wang X., Ma X., Liu J. (2020). Deep learning for edge computing applications: A State-of-the-Art survey. IEEE Access.

[10] Khalil R.A., Saeed N., Masood M., Fard Y.M., Alouini M.S., Al-Naffouri T.Y. (2021). Deep Learning in the Industrial Internet of Things: Potentials, Challenges, and Emerging Applications. IEEE Internet Things J..

[11] Thai-Nghe N., Thanh-Hai N., Ngon N.C. (2020). Deep learning approach for forecasting water quality in IoT systems. Int. J. Adv. Comput. Sci. Appl..

[12] Chen Q., Wang W., Wu F., De S., Wang R., Zhang B., Huang X. (2019). A Survey on an Emerging Area: Deep Learning for Smart City Data. IEEE Trans. Emerg. Top. Comput. Intell..

[13] Syed A.S., Sierra-Sosa D., Kumar A., Elmaghraby A. (2021). IoT in smart cities: A survey of technologies, practices and challenges. Smart Cities.

[14] Warden P., Situnayake D., Loukides M., Taché N. (2019). Tinyml: Machine Learning with Tensorflow Lite on Arduino and Ultra-Low-Power Microcontrollers.

[15] Wang Y., Zhang X., Xie L., Zhou J., Su H., Zhang B., Hu X. (2020). Pruning from Scratch. Proceedings of the AAAI Conference on Artificial Intelligence.

[16] Wardana I.N.K., Gardner J.W., Fahmy S.A. (2021). Optimising Deep Learning at the Edge for Accurate Hourly Air Quality Prediction. Sensors.

[17] Lin J., Chen W.M., Lin Y., Cohn J., Gan C., Han S. (2020). MCUNet: Tiny Deep Learning on IoT Devices. arXiv.

[18] Banbury C.R., Reddi V.J., Lam M., Fu W., Fazel A., Holleman J., Huang X., Hurtado R., Kanter D., Lokhmotov A. (2020). Benchmarking TinyML Systems: Challenges and Direction. arXiv.

[19] TensorFlow Lite. http://www.tensorflow.org/lite.

[20] Dennis D.K., Gopinath S., Gupta C., Kumar A., Kusupati A., Patil S.G., Simhadri H.V. EdgeML Machine LEARNING for Resource-Constrained Edge Devices. https://github.com/Microsoft/EdgeML.

[21] Suda N., Loh D. (2019). Machine Learning on ARM Cortex-M Microcontrollers.

[22] X-CUBE-AI—AI Expansion Pack for STM32CubeMX—STMicroelectronics. http://www.st.com/en/embedded-software/x-cube-ai.html.

[23] Sanchez-Iborra R., Skarmeta A.F. (2020). TinyML-Enabled Frugal Smart Objects: Challenges and Opportunities. IEEE Circuits Syst. Mag..

[24] Puthal D., Mohanty S., Wilson S., Choppali U. (2021). Collaborative Edge Computing for Smart Villages. IEEE Consum. Electron. Mag..

[25] Merenda M., Porcaro C., Iero D. (2020). Edge Machine Learning for Ai-Enabled IoT Devices: A Review. Sensors.

[26] IBM Security (2021). Cost of a Data Breach Report 2021. https://www.ibm.com/downloads/cas/OJDVQGRY.

[27] Page M.J., McKenzie J.E., Bossuyt P.M., Boutron I., Hoffmann T.C., Mulrow C.D., Shamseer L., Tetzlaff J.M., Akl E.A., Brennan S.E. (2021). The PRISMA 2020 statement: An updated guideline for reporting systematic reviews. Int. J. Surg..

[28] Paul A.J., Mohan P., Sehgal S. Rethinking Generalization in American Sign Language Prediction for Edge Devices with Extremely Low Memory Footprint. Proceedings of the 2020 IEEE Recent Advances in Intelligent Computational Systems, RAICS.

[29] Mohan P., Paul A.J., Chirania A. (2020). A Tiny Cnn Architecture for Medical Face Mask Detection for Resource-Constrained Endpoints. Innovations in Electrical and Electronic Engineering.

[30] Coffen B., Mahmud M.S. TinyDL: Edge Computing and Deep Learning Based Real-Time Hand Gesture Recognition Using Wearable Sensor. Proceedings of the 2020 IEEE International Conference on E-Health Networking, Application & Services (HEALTHCOM).

[31] Venzke M., Klisch D., Kubik P., Ali A., Missier J.D., Turau V. (2020). Artificial Neural Networks for Sensor Data Classification on Small Embedded Systems. arXiv.

[32] Orfanidis C., Hassen R.B.H., Kwiek A., Fafoutis X., Jacobsson M. A Discreet Wearable Long-Range Emergency System Based on Embedded Machine Learning. Proceedings of the 2021 IEEE International Conference on Pervasive Computing and Communications Workshops and Other Affiliated Events (PerCom Workshops).

[33] Wong A., Famouri M., Pavlova M., Surana S. (2020). TinySpeech: Attention Condensers for Deep Speech Recognition Neural Networks on Edge Devices. arXiv.

[34] De Prado M., Rusci M., Donze R., Capotondi A., Monnerat S., Benini L., Pazos N. (2021). Robustifying the Deployment of TinyML Models for Autonomous Mini-Vehicles. Sensors.

[35] David R., Duke J., Jain A., Reddi V.J., Jeffries N., Li J., Kreeger N., Nappier I., Natraj M., Regev S. (2020). TensorFlow Lite Micro: Embedded Machine Learning on TinyML Systems. arXiv.

[36] Capotondi A., Rusci M., Fariselli M., Benini L. (2020). CMix-NN: Mixed Low-Precision CNN Library for Memory-Constrained Edge Devices. IEEE Trans. Circuits Syst. II Express Briefs.

[37] Cai H., Gan C., Zhu L., Han S. (2020). TinyTL: Reduce Memory, Not Parameters for Efficient On-Device Learning. arXiv.

[38] LeCun Y., Cortes C., Burges C.J.C. (1998). MNIST Handwritten Digit Database. http://yann.lecun.com/exdb/mnist/.

[39] Tecperson (2017). Sign Language MNIST from Kaggle. Kaggle. https://www.kaggle.com/datamunge/sign-language-mnist/metadata.

[40] Akash (2020). ASL Alphabet from Kaggle. Kaggle. https://www.kaggle.com/grassknoted/asl-alphabet.

[41] Rasband D. (2018). ASL Alphabet Test from Kaggle. Kaggle. https://www.kaggle.com/datasets/danrasband/asl-alphabet-test.

[42] Ashish Jangra (2020). Face Mask Detection ~12K Images Dataset from Kaggle. Kaggle. https://www.kaggle.com/ashishjangra27/face-mask-12k-images-dataset.

[43] Makwana D. (2020). Face Mask Classification. Kaggle. https://www.kaggle.com/dhruvmak/face-mask-detection.

[44] Deng J., Dong W., Socher R., Li L.J., Li K., Fei-Fei L. ImageNet: A Large-Scale Hierarchical Image Database. Proceedings of the 2009 IEEE Conference on Computer Vision and Pattern Recognition.

[45] Bulat A., Tzimiropoulos G. XNOR-Net++: Improved Binary Neural Networks. Proceedings of the 30th British Machine Vision Conference 2019, BMVC 2019.

[46] Imagenet (2018). ImageNet Object Localization Challenge from Kaggle. https://www.kaggle.com/c/imagenet-object-localization-challenge/data%0Ahttps://www.kaggle.com/c/imagenet-object-localization-challenge.

[47] Nilsback M.E., Zisserman A. Automated Flower Classification over a Large Number of Classes. In Proceeding of the 6th Indian Conference on Computer Vision, Graphics and Image Processing, ICVGIP 2008.

[48] Englert B., Lam S. (2011). The Caltech UCSD Birds 200-2011 Dataset.

[49] Parkhi O.M., Vedaldi A., Zisserman A., Jawahar C.V. Cats and Dogs. Proceedings of the IEEE Computer Society Conference on Computer Vision and Pattern Recognition.

[50] Vu T.H., Olsson C., Laptev I., Oliva A., Sivic J. (2014). Food-101–Mining Discriminative Components with Random Forests.

[51] Krizhevsky A. (2009). Learning Multiple Layers of Features from Tiny Images.

[52] Chowdhery A., Warden P., Shlens J., Howard A., Rhodes R. (2019). Visual Wake Words Dataset. arXiv.

[53] Avigan A. (2020). Cleveland Clinic Heart Disease Dataset. Kaggle. https://www.kaggle.com/aavigan/cleveland-clinic-heart-disease-dataset.

[54] Boero L., Cello M., Marchese M., Mariconti E., Naqash T., Zappatore S. (2016). Statistical Fingerprint-Based Intrusion Detection System (SF-IDS). Int. J. Commun. Syst..

[55] Gorman R.P., Sejnowski T.J. (1988). Analysis of Hidden Units in a Layered Network Trained to Classify Sonar Targets. Neural Netw..

[56] Gorman P., Sejnowski T. (1989). Connectionist Bench (Sonar, Mines vs. Rocks) Dataset. UCI Machine Learning Repository. https://archive.ics.uci.edu/ml/datasets/Connectionist+Bench+(Sonar,+Mines+vs.+Rocks).

[57] Loseto G. (2019). Traffic, Driving Style and Road Surface Condition. Kaggle. https://www.kaggle.com/gloseto/traffic-driving-style-road-surface-condition.

[58] EnviroCar—Datasets—The Datahub. http://www.old.datahub.io/dataset/envirocar.

[59] Search for and Download Air Quality Data|NSW Dept of Planning, Industry and Environment. http://www.dpie.nsw.gov.au/air-quality/search-for-and-download-air-quality-data.

[60] Zhang Y., Suda N., Lai L., Chandra V. (2018). Hello Edge: Keyword Spotting on Microcontrollers. arXiv.

[61] STMicroelectronics. https://www.st.com/content/st_com/en.html.

[62] Mahdavinejad M.S., Rezvan M., Barekatain M., Adibi P., Barnaghi P., Sheth A.P. (2018). Machine learning for internet of things data analysis: A survey. Digit. Commun. Netw..

[63] Ian G., Bengio Y., Courville A. (2016). Deep Learning.

[64] Wu Z., Jiang M., Li H., Zhang X. (2020). Mapping the knowledge domain of smart city development to Urban Sustainability: A Scientometric Study. J. Urban Technol..

